# Delineation of *CCDC39/CCDC40* mutation spectrum and associated phenotypes in primary ciliary dyskinesia

**DOI:** 10.1186/2046-2530-1-S1-P91

**Published:** 2012-11-16

**Authors:** M Legendre, S Blanchon, B Copin, P Duquesnoy, G Montantin, E Kott, F Dastot, L Jeanson, M Cachanado, A Rousseau, JF Papon, A Tamalet, AM Vojtek, D Escalier, A Coste, J de Blic, A Clément, E Escudier, S Amselem

**Affiliations:** 1 INSERM, UMR_S933, UPMC Univ Paris 06; and AP-HP, Hôpital Armand-Trousseau, Service de Génétique et d’Embryologie Médicales, F-75012, Paris, France; 2 AP-HP, Hôpital Armand-Trousseau, Unité de Pneumologie Pédiatrique, Centre National de Référence des Maladies Respiratoires Rares, F-75012, Paris, France; 3 AP-HP, Hôpital Saint-Antoine, Unité de Recherche Clinique; and UPMC Univ Paris 06, Unité Fonctionnelle de Pharmacologie, F-75012, Paris, France; 4 AP-HP, Hôpital Inter-Communal et Groupe Hospitalier Henri Mondor-Albert Chenevier, Service d’ORL et de Chirurgie Cervico-Faciale, F-94000, Créteil, France; 5 Hôpital Inter-Communal, Service d’Anatomo-Pathologie, F-94000, Créteil, France; 6 AP-HP, Groupe Hospitalier Necker-Enfants Malades, Service de Pneumologie et Allergologie Pédiatriques, F-75015, Paris, France

## Background

*CCDC39* and *CCDC40* genes have recently been implicated in primary ciliary dyskinesia (PCD) with inner dynein arms (IDA) defects and axonemal disorganization; their contribution to the disease is, however, unknown. With the aim to delineate *CCDC39/CCDC40* mutation spectrum and associated phenotypes, we screened a large cohort of patients with IDA defects, and accurately described their clinical and ciliary phenotypes.

## Methods

All *CCDC39* and *CCDC40* exons and intronic boundaries were sequenced in 43 patients from 40 unrelated families. We recorded and compared clinical features (sex, origin, consanguinity, laterality defects, ages at first symptoms and evaluation, neonatal respiratory distress, airway infections, nasal polyposis, otitis media, bronchiectasis, infertility), ciliary beat frequency and quantitative ultrastructural analyses of cilia and sperm flagella.

## Results

Biallelic *CCDC39* or *CCDC40* mutations were identified in 30/34 (88.2%) unrelated families with IDA defects and axonemal disorganization (22 and 8 families, respectively). Fourteen of the 28 identified mutations are novel. No mutation was found in the 6 families with isolated IDA defects. Patients with identified mutations shared a similar phenotype, in terms of both clinical features and ciliary structure and function. The sperm flagellar ultrastructure, analyzed in 4/7 infertile males, evidenced abnormalities similar to the ciliary ones.

## Conclusions

*CCDC39* and *CCDC40* mutations represent the major cause of PCD with IDA defects and axonemal disorganization. Patients carrying *CCDC39* or *CCDC40* mutations are phenotypically indistinguishable. *CCDC39* and *CCDC40* analyses in selected patients ensure to find mutations with high probability, even if clinical or ciliary phenotypes cannot prioritize one analysis over the other.

